# Somatisation in primary care: experiences of primary care physicians involved in a training program and in a randomised controlled trial

**DOI:** 10.1186/1471-2296-10-73

**Published:** 2009-11-25

**Authors:** José M Aiarzaguena, Idoia Gaminde, Gonzalo Grandes, Agustín Salazar, Itziar Alonso, Álvaro Sánchez

**Affiliations:** 1 San Inazio Health Care Centre, Osakidetza/Basque Health Service, Bilbao, Spain; 2 Continuous Education Unit, Department of Health, Pamplona, Spain; 3 Primary Care Research Unit, Osakidetza/Basque Health Service, Bilbao, Spain; 4 Faculty of Psychology, University of the Basque Country, Donostia, Spain

## Abstract

**Background:**

A new intervention aimed at managing patients with medically unexplained symptoms (MUS) based on a specific set of communication techniques was developed, and tested in a cluster randomised clinical trial. Due to the modest results obtained and in order to improve our intervention we need to know the GPs' attitudes towards patients with MUS, their experience, expectations and the utility of the communication techniques we proposed and the feasibility of implementing them. Physicians who took part in 2 different training programs and in a randomised controlled trial (RCT) for patients with MUS were questioned to ascertain the reasons for the doctors' participation in the trial and the attitudes, experiences and expectations of GPs about the intervention.

**Methods:**

A qualitative study based on four focus groups with GPs who took part in a RCT. A content analysis was carried out.

**Results:**

Following the RCT patients are perceived as true suffering persons, and the relationship with them has improved in GPs of both groups. GPs mostly valued the fact that it is highly structured, that it made possible a more comfortable relationship and that it could be applied to a broad spectrum of patients with psychosocial problems. Nevertheless, all participants consider that change in patients is necessary; GPs in the intervention group remarked that that is extremely difficult to achieve.

**Conclusion:**

GPs positively evaluate the communication techniques and the interventions that help in understanding patient suffering, and express the enormous difficulties in handling change in patients. These findings provide information on the direction in which efforts for improving intervention should be directed.

**Trial registration:**

US ClinicalTrials.gov NCT00130988

## Background

General practitioners play a pivotal role[1,2] in managing one of the most complex problems encountered in modern medical practice: the large number of patients with somatisation disorders in primary care[3]. While some interventions have proved effective at a specialised level and while there are many recommendations for managing patients with medically unexplained symptoms (MUS)[4], GPs often feel blamed for their poor results in managing patients with mental illness and also feel that they are unable to do so and have emphasised the sense of frustration, anger and powerlessness in the face of patients with persisting somatising symptoms[5].

The sparse research in the field of somatisation in primary care has tended to focus mainly on whether interventions serve the needs of patients, without taking into account either the GP's opinion about the intervention itself, or about the difficulties that GP's have to face in order to implement those interventions. Nevertheless, GP's attitudes, experiences and expectations are also important, since they are essential to the successful implementation of any method for managing MUS in primary care. It is therefore necessary to ascertain what is most valued by GPs about interventions recommended for MUS patients.

Our team has developed an intervention for MUS patients based on a specific set of communication techniques that has been tested through a randomized controlled trial [6]. In the trial, one group of patients was treated by GPs with a minimum of training in Goldberg's reattribution techniques and the other group was treated by GPs who received a more intensive training that included specific instructions in the use of a standardized set of communications techniques.

In order to improve our intervention we need to know the GPs' attitudes about patients with MUS, their experience, expectations and the utility of the communication techniques proposed by us and the feasibility of implementing them.

## Methods

All GPs (n = 39) from the Basque Health Service who had taken part during the previous year in a Cluster Randomized Controlled Trial to assess the effectiveness of a short psychosocial intervention for somatising patients were invited to participate in this investigation. The cluster randomised controlled clinical trial was approved by the Clinical Research Ethics Committees of the participating centres and was registered on ClinicalTrials.gov on August 16, 2005 (NCT00130988). The informed consent of both doctors and patients was obtained. Fifty percent of doctors were women and on average they were 42 years old, and accounted for 13 years of clinical practice experience in primary care.

GPs assigned to the comparison group ('active control group') provided the 'best standard of care' based on the reattribution of symptoms. They were trained to emphasise a link between symptoms and emotions[7,8] Total duration of training was 3 hours which was less than the 8 hours recommended by Goldberg and colleagues [7] because of the previous background experience of the participating GPs. The treatment manual for this group included examples for the articles of Mather and Gask[8] and Goldberg[7] on how the authors performed validation of symptoms and established a link with the psychosocial problems detected. In contrast, GPs assigned to the intervention group were trained: 1) to explain symptoms to the patient in a physical and tangible manner as resulting from hormonal imbalance[9,10]; 2) to explore psychosocial aspects through an indirect approach; 3) to attribute hormone release to irrational thoughts; and 4) to 'normalise' the patients' symptoms, understood as conveying to the patient that anyone with the same symptoms or under the same circumstances would feel exactly the same way[6,11]. Duration of training was 20 hours divided into five 4-hour sessions over two and a half days. In the first session, the relevant standardized communication techniques were described and presented as an effective tool for resolving GPs' antipathy towards somatisers, patients' fears and the most likely points of confrontation with patients with medically unexplained symptoms. This session included a short theoretical lecture followed by small-group discussion and common agreement among the groups. The purpose of the remaining four sessions was to train GPs in communication skills through role-playing, with pairs taking turns in being "doctor" and "patient".

Health-related quality of life (assessed with the 36-item Short-Form Health Survey, SF-36) was used as outcome measure, and the communication techniques were found to have a clinically relevant impact on body pain and a trend towards better scores in the remaining scales.

A qualitative study with four focus groups was conducted at the end of the trial, with both researchers and GPs blind to the outcomes of the trial[12]. Focus groups were scheduled separately, two for physicians in the active control group (ACG) and another two for physicians in the new intervention group (NIG). Participation rate was 67% (26 out of 39): 70% (14/20) from the ACG and 63% (12/19) from the NIG. All groups met in the library of the Bizkaia Research Unit. All focus groups were aided by two of the authors (IG and IA) with different backgrounds (sociology and psychology). Each session began with introductions and a brief explanation of the reasons for the investigation and of its confidentiality. The same set of questions was posed to each group:

• What motivated you to participate in this research project?

• What did you hope to gain from the intervention?

• What were your expectations; was this achieved?

• Were your expectations fulfilled?

• Has your clinical practice with regard to this type of patient changed?

• Which aspect of the intervention do you consider the most relevant;

• Do you think that your perception of somatising patients has changed?

• Do you see a marked difference between your relationships with somatising patients before and after your participation in this trial?

• What are the differences;

• How was the experience of being in the control group for you?.

Participants were encouraged to talk freely and, if they brought up relevant points spontaneously, the order of questions was varied to maintain the flow of the session.

Focus groups lasted for approximately 40 minutes in the active control group and about 90 minutes in the new intervention group. Interviews were tape-recorded and transcribed verbatim. This paper is oriented to providing thorough descriptions and interpretations of the research aims - guided by the interview questions, including the meaning it holds for those who experience it, rather than theory building. IG and IA first went through the transcripts, reading and annotating them to gain an insight into the data. Following Bloor[12] the first step was to index the data in order to make it manageable for interpretation. The aim of indexing is to bring together all extracts of data that are pertinent to a particular theme, topic or hypothesis. The process of indexing then involves the analyst reading and re-reading the text and assigning index codes, which relate to the context of the data and are of interest to the research analytical framework. Based on these themes, patterns were identified and coded. This coding exercise required several readings through the transcripts as categories of topics evolve; later each piece of coded material was grouped under a specific category or subcategory. During the indexing of the focus groups data, analysts ensured that the context of any speech extract was studied, looking at any one individual's speech over the course of the focus group and looking at how the speech fits into what other participants are saying. With these categories and subcategories and their relationships, a conceptual framework was devised and applied systematically to the data by the whole research team.

## Results

Both groups GPs stated that after their participation in the clinical trial, they perceived the patients as true suffering persons; their relationship with the patients had improved, although there continued to be complicated cases and the doctors still preferred attending to other types of patient.

### Reasons for taking part in the training program

Doctors fundamentally gave two reasons for agreeing to participate in the study. Firstly, the necessity of finding a useful tool to help them work with this type of patient, characterised as complicated and problematical, and secondly, confidence in the research team.

*'Personally, what brought me here was to see whether I could learn something about how to manage these patients. These patients are a real problem in my surgery really, so I thought, let's see what they're offering, whether there's anything... here that can be used, (I wanted) to learn how to deal with this problem better, that's all' (NIG1)*.

*'For me specifically, it was the researchers, in other words the, well... more than being a question of motivation, for me it was one of participating, of collaborating with a colleague, José Mari Aiarzaguena, who was planning a project that I thought was serious - that was really my only motivation' (ACG2)*.

*'I have the problem, but no answer' (ACG1)*.

### Benefits

1. The intervention is clearly structured.

Physicians assigned to the intervention group rated the experience positively mainly because it provided them with a road map, a guiding line or frame of reference in their surgeries. *"So, as well as having a protocol for hypertension, or a problem, isn't that so? I know that... now we've also got a method, so the consultation appears to have a thread, a connecting thread, doesn't it? (NIG1)*

*'Before, when you wanted to enter the psychosocial world, the consultation turned into a chat session, you never knew where you were going and the... the fact of participating here gives you... at least it gives you a road map...' (NIG2)*.

2. Participation in the training afforded doctors in both groups a better understanding of somatising patients. It facilitates a more comfortable relationship.

Both groups highlight this as a positive point. Somatising patients are difficult to deal with and can elicit contempt, but after spending time with them doctors become aware that they are people who are suffering and have problems. Despite this, they still prefer to attend to other types of patient.

*'I understand them better, I now believe these patients and I also believe in all the... in all this somatic suffering that they have, right? I mean I understand them, whereas before I saw them as real fakers' (NIG2)*.

*' ... I think that it's a question of valuing them for what they are. I mean, and maybe I'm going to exaggerate here, but before the trial, I'm going to exaggerate, eh? They were annoying - what I wanted was to get rid of them - so with a new focus, something which would never have occurred to me, I value them more as persons, not only as patients [...] I'm more comfortable in the relationship' (NIG2)*.

*'Because they are people that seemed annoying before, and obsessive, and now having an explanation for the whole cycle, I want to say that... that there is something that makes you understand all these people a bit more' (ACG1)*.

Since the trial, they are less afraid of consultations with somatising patients, due to the fact that they have a better relationship with the patients.

*'Change is something that has taken place in us, more than in the patients' (NIG2)*.

*'I feel more comfortable taking care of them [...] communication with these patients has clearly improved, there is more empathy' (NIG2)*.

3. It may be used with other patients with psychosocial problems, not only patients with medically unexplained symptoms.

This opinion was only expressed by the doctors in the intervention group. It seems that once the tool has been learned, it is then useful for attending to grieving patients, or those suffering from depression or anxiety. The doctors suggest using specific aspects of the DEPENAS proposal.

'*It has even changed how I work with grieving patients, for example, support, and little things that, without applying the complete, systematic Depenas method; it's enough to apply some of the things that we have learned in the training, isn't that right? And wham! You know that it... that it works, or at least that it is... that it's gratifying...' (NIG1)*.

*'Personally, I believe that for seventy percent of my cases, it could be an extremely useful technique' (NIG2)*.

### Limits and barriers

1. Patient's change is more complicated than previously thought

Doctors from both groups consider that change on the part of the patients is necessary (that they learn to put things in perspective, look after themselves, etc.), but that it is extremely difficult to achieve. Those who took part in the intervention group put a higher priority on their feeling comfortable with the relationship themselves, than on patient results.

*'I don't know, the change that, that we were aiming for, right? Or that we... it's not, it's not clear to me that this was achieved in my patients' (NIG1)*.

*'Almost nobody wants to change. I mean, if you can change them yourself, without them making an effort, that's wonderful; but a personal effort from them... that's difficult, very difficult' (NIG2)*.

*'We still don't know how it might have affected the patients, but for me, brilliant... If on top of that the results are good then... then that's the icing on the cake' (NIG2)*.

2. The shortage of Time is still a problem in primary care consultations

Both groups mentioned the amount of time that they needed to dedicate to these patients. They raised the question of whether, if this is a common problem in primary care, it makes sense to dedicate so much time to this type of patient, given that they perceive the patient care situation as being under pressure. This led into a debate over which type of patient *should *be given time. Why this time should not be dedicated to somatising patients? Why are they considered as a different category of patient, with differing needs from those of hypertensive patients, diabetics, etc.? The GPs considered the time required for managing them to be beyond their capacity in primary care consultations.

*'Why should we give priority to hypertensive patients, just because they are hypertensive, and not to those people for whom unhappiness is the underlying cause of it all? So it doesn't seem to me that we are giving them priority; but now I will be able to say to them that you are in the same category, or have the same rights as someone who comes in with a cold, or for haemorrhoids, or with hypertension...' (ACG2)*.

They are already considered by the doctors as people with the same rights as those whose symptoms can be explained medically - as we mentioned above - but both groups concur in stating that the time that they have to dedicate to them is excessive and it is apparent that this technique may not be applicable outside the investigation, in everyday clinical practice.

*'Well, it's all the same whether instead of five half-hour sessions... all the same with one or two sessions, it could still be enough; I mean, there's no need for five sessions, is there? Or however many there are' (NIG2)*.

*'As a protocol it's very long, I mean in time, not in importance, in time' (NIG2)*.

3. Medical training and its usefulness in primary care

In the interviews, doctors in both groups raise topics related to general practitioner training and a lack of relevant training. *'We are neither psychiatrists, nor psychologists... but family physicians, we need answers to give these patients' (ACG2)*.

Finally, participants in both groups affirm that their relationships with these patients are now easier and that they consider their participation in the study to have had positive effects.

## Discussion

Our investigation agrees with other studies that the reasons for doctor participation were the necessity of finding a useful tool for managing these patients, whom they consider as difficult, and confidence in the research team[13]. All participants in the focus groups state that they have become closer to this type of patient and that their perception of them has changed. This may be due to the central role that training was given in the investigation, as GPs find that training improves their ability to cope with difficult patients[14]. Yet, they still perceive these patients as complicated [15].

GP's changed their feeling and perception of MUS patients after participating both in training and clinical trial. Patients stopped being a nuisance in their surgeries, and became perceived as true sufferers, so that their use of the health services became understandable as well. Nevertheless, GPs preferred not to attend to them because they did not know how to treat them. This change suggests a step forward towards the bio-psycho-social model. The words used to describe these patients (patient with medically unexplained symptoms) clearly points out that these patients are excluded from the medical model. We support Fink and Rosenthal's proposal of using the term functional disorders [16]. One of the main aims of the use of a specific set of communication techniques was to get a better knowledge of the patients. But we do not know whether the change of attitude towards them is due to the specific training, to time dedicated to these patients or to both factors. It should be borne in mind that doctors spend on average six sessions of more than half an hour with each patient.

GPs trained in the new intervention were more likely to report that they felt more comfortable in their interaction with patients. However, they were also more likely to report difficulties encountered in managing the change needed in these patients. This suggests that the set of communication techniques they were trained in to facilitate a collaborative relationship worked; whereas the strategy tested to manage change -based in the attribution of irrational thoughts to a hormonal imbalance- has not worked, and needs a fresh approach. GPs now are well aware of patients' suffering, but would prefer to care for another sort of patients.

We were also able to determine which elements of the intervention were most highly valued by the GPs taking part in the investigation. For those in the intervention group, the key elements of the intervention were identified as: its structure, because it facilitates a more comfortable relationship, and the fact that it can be applied to a broader spectrum of patients with psychosocial problems.

In a similar study Dowrick[17] conducted a questionnaire survey and undertook qualitative interviews with GPs who were taking part in an exploratory RCT of reattribution training. Their results related to benefits were similar to ours, i.e. doctors valued positively a better understanding of MUS patients, the additional structure provided by reattribution, and the use of reattribution in their consultations with non-MUS patients. On the contrary in our study only time was mentioned as a barrier. Probably because Dowrick's study tested the feasibility of the implementation of reattribution in everyday clinical practice, and focused on patient barriers, doctor barriers, consultation barriers, diagnostic barriers and barriers in the healthcare context. In our study doctors received trained and took part in a clinical trial, i.e. having to treat 4 patients -selected randomly- in a standardized way. Hence, they focused on the limits and barriers encountered in the intervention with MUS patients, and mainly stressed the problems they encountered in dealing with patients' change.

Time spent with patients in the clinical trial was considered excessive for application in the highly congested primary care environment. This opinion, which is widely held amongst physicians, merits two considerations, some of which were suggested by the focus groups. Firstly, somatising patients are frequent users, so it may be more appropriate to talk about better management of the time that is already used extensively by them, i.e. optimising the time taken up by patient-requested consultations, by having them scheduled by the physician. Moving from patient-led to doctor-led consultations. Secondly, a conceptual issue arises: rather than defining patients only in terms of their use of services as frequent versus occasional attenders, their needs should be taken into account when discussing appropriate use of health care[18]. The 156 somatising patients in the clinical trial had presented, on average, 15 active symptoms during the year prior to the study, in association with mental illness in 80% of patients, and with a health-related quality of life measured by SF-36 of two standard deviations below the average of the general population. Therefore, the question we would like to pose is: how should one define an appropriate use of consultation time in primary care by patients with so many symptoms and such a poor quality of life? Is it appropriate to try to reduce the use of services by patients with MUS, whether this is not a consequence of a reduction in their health needs or, which amounts to the same thing, an improvement in health-related quality of life?

### Strengths and limitations

GPs in this investigation had all participated in the clinical trial and many of them had received prior training in clinical interviewing and mental health. They may therefore be taken as a group of doctors who tend to be well disposed towards the implementation of psychosocial interventions within routine clinical practice in primary care. For this reason they may be considered as 'experts' and their opinions could therefore be useful when drawing up a psychosocial intervention for use in primary care. We see this as a major strength of this study.

The main limitation of this study is that the views of these GPs cannot be assumed to be representative of the whole group of primary care physicians. The fact that the topic guide did not have negatively framed statements could lead participants towards more positive comments; and, unfortunately, we did not check findings with focus group participants. Although GPs' reasons for not participating in focus groups were due to prior engagements, we cannot rule out that their perception was more negative.

## Conclusion

Both communication techniques and interventions oriented towards patients' suffering change GPs' feelings towards these patients and improve the quality of their interaction. GPs highly value that the intervention tested could be used as a framework that can be useful with patients with psychosocial suffering -not just somatizers- and that it proposes strategies to promote change in patients. But, although the latter did not prove efficient in the trial, it is the key that should guide the efforts to improve intervention.

## Competing interests

The authors declare that they have no competing interests.

## Authors' contributions

JMA conceived the study, participated in its design, coordinated and helped to draft the manuscript. IG participated in the design of the study, moderated and analysed the focus groups and helped to draft the manuscript. GG conceived the study, participated in design of the study and helped to draft the manuscript.

AS helped to draft the manuscript. IA moderated and analysed the focus groups and helped to draft the manuscript. AS helped to draft the manuscript. All authors read and approved the final manuscript.

## Pre-publication history

The pre-publication history for this paper can be accessed here:

http://www.biomedcentral.com/1471-2296/10/73/prepub
